# Development of a phantom‐based end‐to‐end QA workflow for online adaptive radiotherapy incorporating high‐resolution 2D detector measurements

**DOI:** 10.1002/acm2.70898

**Published:** 2026-11-03

**Authors:** Julian Niedling, Robert Schindhelm, Luis Cervantes, Gary Razinskas, Sonja Wegener

**Affiliations:** ^1^ Department of Radiation Oncology University Hospital Würzburg Würzburg Germany; ^2^ IBA Dosimetry GmbH Schwarzenbruck Germany

**Keywords:** 2D Measurements, End‐to‐End Workflow, Gamma Analysis, Online Adaptive Radiotherapy, Quality Assurance, Varian Ethos

## Abstract

**Background:**

Online adaptive radiotherapy (oART) accounts for anatomical changes by adjusting treatment plans on a daily basis. However, comprehensive plan‐specific quality assurance (QA) is challenging, as pre‐treatment measurements cannot be performed with the patient on the treatment couch. Therefore, an end‐to‐end QA approach, covering imaging, contouring, plan adaptation, dose calculation, and delivery, is essential to ensure accurate and reliable oART.

**Purpose:**

To develop and evaluate an end‐to‐end QA workflow for oART, incorporating high‐resolution detector‐based 2D dose verification and interchangeable phantom inserts.

**Methods:**

A novel QA workflow was implemented using the myQA® SRS phantom with a 2D CMOS detector and a 3D‐printed anthropomorphic pelvis insert. A virtually deformed planning CT was generated to simulate anatomical changes and used as input for the adaptive workflow. The process included CBCT acquisition, manual contour adaptation, plan generation, dose delivery, and comparison of measured and calculated dose distributions via Gamma Analysis. Workflow feasibility was tested through repeated measurements. Error detectability was evaluated by altering gantry/collimator angles, monitor units (MU), phantom positioning, and CBCT presets. Confidence limits, calculated following AAPM TG‐119 methodology, were used as plan‐specific thresholds for evaluating Gamma Pass Rates (GPR). Corresponding dose‐volume histogram (DVH) parameters, using D98% for the planning target volume (PTV) and V95% for organs at risk (OARs), were calculated.

**Results:**

Ethos auto‐contouring did not perform reliably on the phantom geometry, necessitating manual contouring. All workflow repetitions produced high GPR values for 3 %/2 mm, 2 %/2 mm, 3 %/1 mm, and 2 %/1 mm global criteria between measured and calculated dose. Confidence limits served as effective thresholds for identifying plan deviations. Introduced errors, including gantry angle deviations > 3° and collimator angle deviations ≥ 3°, MU variations ≥ 3 %, incorrect CBCT presets, and 3 mm setup shifts, were clearly detectable across all criteria. Smaller deviations, such as 1° angular changes or 1 % MU variation, resulting in minor dosimetric effects residing at or below 1.2 % for the PTV and 5.6 % for OARs, yielded GPR values above the Confidence limit.

**Conclusions:**

The proposed workflow is feasible, reproducible, and capable of reliably identifying plan‐specific deviations in Ethos oART. High‐resolution 2D measurements and dose comparison provided robust verification of adaptive dose calculation and delivery. While limited by manual contouring and data‐handling constraints inherent to the Ethos platform, this method represents a practical end‐to‐end QA approach for clinical oART implementation.

## 1 INTRODUCTION

Anatomical changes such as variations in size, shape and position of organs during the course of radiation therapy can be compensated for in online adaptive radiotherapy (oART) by adjusting the radiation plan on a daily basis.^1^, ^2^, ^3^, ^4^ Since plan‐specific quality assurance (QA) cannot be performed using pre‐treatment measurements while the patient is positioned on the couch, a practical approach for comprehensive, measurement‐based verification of the adaptive workflow is still lacking.^1^, ^5^, ^6^, ^7^, ^8^ Acknowledging that plan‐specific measurement‐based QA is not always feasible for every adapted plan, Chetty et al. (2024, ASTRO White Paper) nevertheless emphasizes that a comprehensive QA program for oART should include end‐to‐end testing that spans image acquisition, delineation, plan adaptation, dose calculation, and dose delivery, ideally validated through independent measurements. In addition to common QA tasks not required for any linac, particular attention should be given to image quality, robust treatment planning, image registration, and dose accumulation.^9^

To support verification within the adaptive workflow, several QA concepts for oART have been proposed. Maraghechi et al. (2022) developed a phantom‐based QA method based on an anthropomorphic pelvis insert and electron density phantom for verifying the dose‐accumulation functionality of the Varian Ethos system by comparing manually summed doses with system‐generated accumulated doses.^10^ However, this approach focused primarily on verifying dose accumulation using virtual deformations and did not incorporate independent detector‐based dose measurements. Chapman et al. (2022) evaluated the robustness and reproducibility of the AI‐assisted online segmentation and adaptive planning process on the Varian Ethos system, using deformed images of an anthropomorphic pelvis phantom to simulate multiple virtual anatomical scenarios.^11^ Their analysis emphasized contour similarity and dose–volume agreement rather than direct dosimetric measurement. Neither of these studies incorporated independent detector‐based dose measurements. Yoganathan et al. (2025) introduced an end‐to‐end QA procedure for the Varian Ethos system that integrates deformed CT datasets of phantoms with detector arrays such as the OCTAVIUS‐4D, ArcCHECK, and RUBY head phantom to evaluate the consistency between planned and measured dose distributions.^12^

Existing concepts lack the combination of realistic anatomical representation and high‐resolution measurements for clinically relevant body regions, particularly the pelvis. While Yoganathan et al. (2025) incorporated both high‐resolution measurements and interchangeable inserts into their procedure, these features were evaluated separately rather than simultaneously. An insert‐based design, however, offers the potential to unify both capabilities within a single, integrated platform. As part of the present study, we implemented and evaluated a novel end‐to‐end workflow designed to address key limitations of existing concepts, particularly the lack of high‐resolution, detector‐based verification combined with the lack of interchangeable inserts tailored to daily adaptive plans. Dose calculation and delivery for oART on the Ethos 2.0 System with HyperSight (Varian Medical Systems, Palo Alto, USA) were verified using 2D dose measurements. For this purpose, the myQA SRS phantom (IBA Dosimetry, Schwarzenbruck, Germany) with a slot for interchangeable inserts was used in combination with an anthropomorphic pelvis insert (PhantomX, Berlin, Germany). A virtually deformed CT, generated from the SRS phantom and insert geometry, was used to emulate anatomical changes and to support adaptive plan generation. High‐resolution 2D measurements were acquired using the myQA SRS 2D Complementary Metal Oxide Semiconductor (CMOS) detector array, enabling a Gamma assessment.

First, an overview of the phantom, measurement equipment and planning systems required to perform the QA workflow is provided. Furthermore, a detailed description of the QA workflow, including step‐by‐step instructions for its execution on the Ethos system combined with a representative clinical application to support workflow traceability, is presented. As Ethos AI‐assisted segmentation plays an important role in the QA workflow, its performance on the phantom is examined within the context of the workflow in the results section. The feasibility and practicability of the proposed end‐to‐end QA workflow were assessed through Gamma Pass Rate (GPR) analysis of six consecutive executions of the adaptive workflow. Finally, the workflow's capability to detect plan‐specific errors was evaluated using GPRs determined with different Gamma criteria.

The aim of this study was to implement an end‐to‐end QA workflow for the Ethos oART system and to demonstrate both its feasibility and capability to detect potential plan‐specific errors using GPR analysis.

## 2 MATERIALS AND METHODS

This section provides a comprehensive overview of the proposed end‐to‐end QA workflow for Varian Ethos linear accelerators (linacs), as well as the required phantom and measurement equipment.

### 2.1 Phantom and measurement equipment

For this study, a 3D‐printed, unscaled anthropomorphic insert (Figure 1a), representing a segment of male pelvic anatomy was manufactured by PhantomX GmbH (Berlin, Germany). It was based on a full pelvis CT (“Full pelvis CT”) and designed to fit the SRS phantom slot. The SRS phantom is a cylindrical body constructed from water‐equivalent material (RW3, density 1.045 g/cm^3^) with a rotatable slot (approximately 4 cm × 15 cm × 15 cm) aligned along the longitudinal *Y*‐axis. “planCT” shows the CT of the pelvis insert positioned inside the SRS phantom, while “planCT*” depicts the virtually deformed insert anatomy, as further described in Section 2.3. For CT imaging at 120 kV on a SOMATOM go.Open Pro (Siemens Healthineers, Erlangen, Germany) with Qr40 reconstruction kernel, the measured HU (mean ± standard deviation) of the prostate and organs at risk were as follows: prostate, 54 ± 15 HU; bladder, 22 ± 16 HU; and rectum, 20 ± 49 HU. These values are consistent with real pelvic anatomy,^13^ thereby enabling representative plan calculation.

**FIGURE 1:**
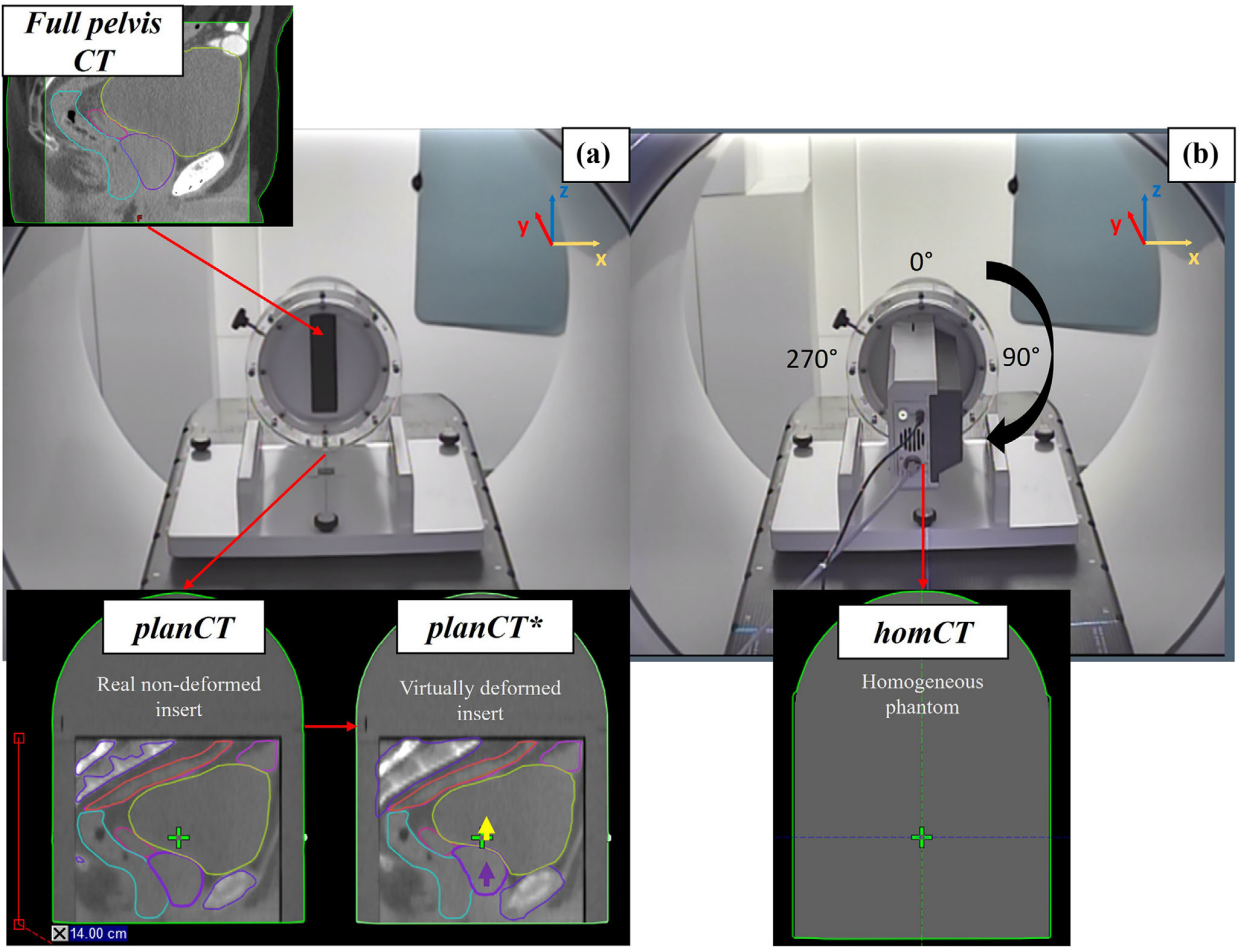
(a) 3D‐printed pelvis insert (black cuboid) based on the full CT scan is entered into the myQA SRS phantom and rotated clockwise to 270° (detector array surface and insert orientation was 0° when facing upwards). The CT image of this setup is shown as “planCT”, including registered contours from the original full pelvis CT (“Full pelvis CT”—green box shows registration window). The planCT is virtually deformed (“planCT*”) in preparation of the proposed end‐to‐end test. Plan adaptation is performed with this setup. (b) Before irradiation of the adapted plan, the pelvis insert is replaced by the myQA SRS detector. The virtual CT (“homCT”) represents the homogeneous phantom geometry used for the comparison of measured and calculated dose.

To demonstrate the feasibility of the proposed end‐to‐end QA workflow, the SRS detector array (ABS, density 1.040 g/cm^3^), which provides a spatial resolution of 0.4 mm and a sensor area of 12 × 14 cm^2^, was positioned in the phantom slot and rotated to 270° (Figure 1b). “homCT” illustrates a vendor‐provided virtual CT representing the homogeneous SRS phantom geometry with 45 Hounsfield Units (HU), which is in accordance with vendor specifications for the used phantom. This data set is used for dose calculation and measurement comparison. All measurements were acquired and analyzed using the dedicated myQA software (IBA Dosimetry, SW Version: 2.18, Release: 2023‐001).

### 2.2 Linac and planning systems

The study was conducted using the Ethos System, an adaptive radiation therapy platform comprising a linac and 3D kV Cone Beam Computed Tomography (CBCT) imaging with HyperSight, which provides HU accuracy comparable to conventional planning CTs, optimizing the adapted plan directly on the CBCT. The software used was Ethos Treatment Management (ETM) 3.0 (full version 03.00.10) and Ethos Treatment Planning (ETP) 2.0. The Ethos linac delivers a 6X‐FFF beam with a dose rate of 600 monitor units (MU) per minute and 1 cGy/MU at a calibration depth of 10 cm, using a dual‐layer multileaf collimator. In addition, Eclipse TPS (Version 18.0, Varian Medical Systems, Palo Alto, USA) was employed for treatment series assignment and data exchange within the workflow, as described below.

### 2.3 End‐to‐end QA workflow

#### 2.3.1 Overview

Figure 2 provides an overview of the workflow and the required steps: For initial setup of the end‐to‐end test, a planning CT of the SRS phantom with the pelvis insert was acquired once. This CT was deformed virtually in order to mimic inter‐fractional anatomical changes between the planning CT and the treatment‐session CT. This enabled using only one physical pelvis insert during the adaptive session, preserving the possibility to mimic multiple anatomical changes by introducing different deformations. The repeated measurement procedure of the proposed end‐to‐end test follows the typical online adaptive workflow, which consists of three main steps. In a first step, the pelvis insert is entered into the SRS phantom, imaged in the Ethos system, and contoured.

**FIGURE 2:**
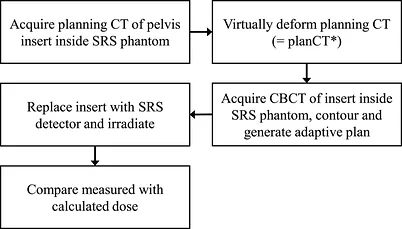
Workflow overview.

An adaptive treatment plan is generated. In a second step, the pelvis insert is replaced by the SRS detector array. The adapted plan is irradiated and the resulting dose distribution is measured. In a third step, the measured dose is compared with the calculated dose.

While the general concept of the end‐to‐end test is straightforward and steps 1 and 2 follow the clinical workflow, the initial setup of the treatment plan and the dose comparison in step 3 require a series of actions and the creation of multiple datasets (patients) due to the limited possibilities within Ethos Treatment Management, necessitating the use of Eclipse as a second treatment planning system for certain preparatory tasks. An overview of the workflow is shown in Figure 3. For workflow reproducibility, all steps are described in more detail in the following paragraphs.

**FIGURE 3:**
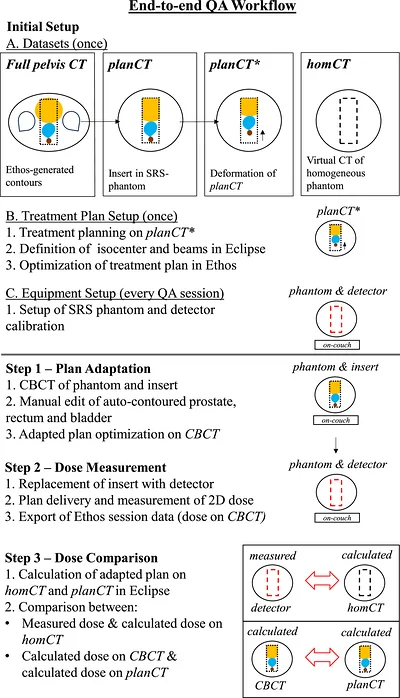
Description of the end‐to‐end QA workflow, comprising the initial setup (datasets, treatment plan and equipment) and repeated workflow steps 1–3.

#### 2.3.2 Initial setup

##### Datasets

In the initial setup, four individual datasets representing different phantom configurations, as described in detail below, were created in the Eclipse TPS. These separate datasets were required because the Ethos system manages image datasets and associated plans within fixed reference frames, and dose calculations must be performed within the corresponding image geometry. Datasets entered in Eclipse become available in the Ethos system automatically.

The original full pelvis CT (“Full pelvis CT”) dataset comprised the original comprehensive pelvic CT provided by the manufacturer (PhantomX). This scan represented a male pelvis anatomy with bowel contrast medium, on which the Ethos system successfully generated automated contours for the prostate, bladder, rectum, and external body. Since the Ethos auto‐contouring algorithm was restricted by the anatomical features embedded within the insert of the otherwise homogeneous phantom, the automated contours were transferred from the full pelvic dataset to the planCT.

For the planCT, the SRS phantom with the anthropomorphic pelvis insert was imaged. The insert was inserted into the slot and rotated to an orientation of 270° (vertical) on the SRS phantom, positioning the insert anatomy head‐first, supine. Subsequently, radiopaque markers were placed at the SRS phantom's marking lines to define a reference point, which was later used as the isocenter for the planCT during treatment planning. The planCT was acquired using the pelvis preset (120 kV) on a SOMATOM go.Open Pro (Siemens Healthineers). The full pelvis CT and the planCT were rigidly registered within Eclipse and contours from the full pelvis CT dataset were copied to the planCT and cropped to match the dimensions of the insert.

A virtually deformed planning CT, called planCT* in the following, was generated. To this end, a custom‐developed deformation tool was used to generate a 3D deformation vector field (DVF). The DVF was created using a custom Python script that used the prostate segmentation as a spatial reference to define the region subjected to deformation, applying a localized displacement to the corresponding voxels with smooth transitions at the deformation boundaries to avoid abrupt tissue warping. This DVF was then imported into the open‐source medical image analysis platform 3D Slicer,^14^, ^15^ where it was applied to both the planCT and its contours. For this study, an exemplary anatomical deformation was applied, consisting of a 10 mm cranial translation of the prostate toward the bladder with rectal distension and bladder compression^16^ (Figure 4). This deformation required corresponding contour adjustments for the prostate, bladder, and rectum.

**FIGURE 4:**
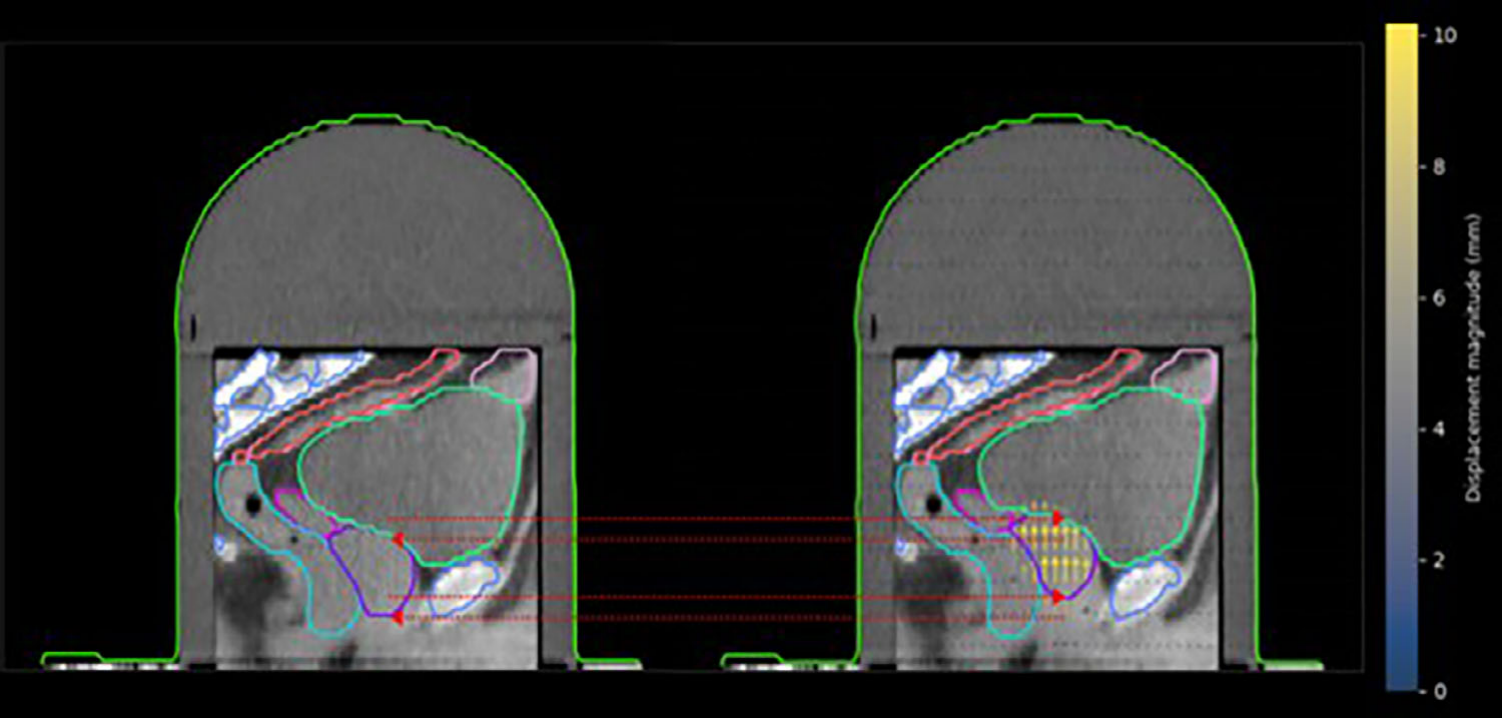
Sagittal CT views of the pelvis insert inside the SRS phantom. Left: original anatomy with contours. Right: deformed CT and contours (prostate 10 mm translation cranial, indicated by red lines); the DVF is shown (yellow arrows) and the colour scale indicates displacement magnitude.

The resulting DVF was reviewed using Jacobian determinants throughout the deformation field, which indicated the absence of tissue folding, and by visual inspection of the resulting anatomy. The propagated contours showed the expected displacement and remained anatomically consistent following the transformation.

A further dataset contained a virtual CT of the homogeneous phantom geometry, called homCT in the following, for the purpose of comparing measurements using the detector array against a calculated reference.

##### Treatment plan setup

Treatment planning was performed on the planCT*. A prostate RT‐intent, along with corresponding planning instructions, was assigned to this dataset. A prescription of 2.75 Gy per fraction was chosen, consistent with the protocol of the Systemic Therapy in Advancing or Metastatic Prostate Cancer: Evaluation of Drug Efficacy Arm H (STAMPEDE‐H) trial.^17^

For a defined position of the isocenter, a radiotherapy plan template was created in Eclipse TPS. For this plan, the isocenter was aligned with the radiopaque isocenter markers indicating reference point in the phantom. The beam geometry was an Intensity Modulated Radiotherapy (IMRT) plan with nine coplanar, equidistant fields starting at a 180° gantry angle, using a fixed collimator angle of 10°. The plan was optimized in Ethos and clinically and technically authorized.

##### Equipment setup

Preparations in the myQA software and detector were required once before every QA session with the SRS phantom placed on Ethos couch: After background signal measurement and gantry angle sensor setup, the detector was placed in the SRS phantom slot at 0° position and calibrated by irradiating a 10 × 10 cm^2^ field with 100 MU, using the corresponding, known calculated dose. Subsequently, the detector was removed and replaced with the pelvis insert and rotated to a 270° position for this study.

#### 2.3.3 Step 1–plan adaptation

At the linac, the phantom including the pelvis insert was aligned with the treatment isocenter, then imaged using the pelvis CBCT preset (125 kV). Proposed contours of the prostate, rectum and bladder were reviewed and manually adjusted, according to the actual insert geometry. An adapted plan (RTPlan), optimized on the CBCT, was created by Ethos and selected for irradiation. Prior to delivery, the pelvis insert was removed and replaced by the SRS detector.

#### 2.3.4 Step 2–dose measurement

The SRS detector measurement was acquired during irradiation of the adapted plan, and the result was automatically saved as a 2D dose grid in the myQA software. Following irradiation, session data were exported locally to access the radiotherapy dose (RTDose) calculated on the CBCT within the Ethos ETP for further comparison.

#### 2.3.5 Step 3–dose comparison

The detector measurements had to be compared to the dose distribution calculated on the detector geometry. Additionally, to cross‐check the calculated dose distribution on the CBCT, the dose distribution on the planCT had to be calculated. Hence, the RTplan from the adaptive session, was calculated both on the planCT and the homogeneous phantom dataset homCT.

To enable re‐import and dose calculation within Ethos ETM, several steps in the Eclipse TPS were required. Initially, the irradiated plan was automatically transferred to the corresponding Eclipse treatment series. However, for plan import, Ethos ETM requires the same plan reference frame as in Eclipse TPS. Structure sets of the planCT and the homCT were duplicated and added to the irradiated plan. Missing couch structures were added. The target volume was coarsely delineated at the prostate location, which was solely required to pass Ethos plan validation. *X*, *Y* and *Z* isocenter coordinates were set to 0.00 cm for all fields, and the volume dose was recalculated to apply isocenter and MU values to the copied plan. The RTPlans were then exported locally from Eclipse and imported to Ethos ETM, conserving MU per field. To obtain a finer dose grid resolution of 1.25 mm (instead of 2.5 mm), the high‐fidelity mode was selected in Ethos. RTDose distributions of the imported RTPlans were calculated by Ethos and exported.

The exported RTPlan and RTDose files from the planCT and homCT were imported into the myQA software. To match the detector's orientation during measurement, the Dose Plane Extraction was set to 270° in the myQA software. The dose distribution on the CBCT measured in step 2 was compared with the calculated dose distribution on the homCT using Gamma Analysis. To maintain workflow integrity, no manual spatial alignment was applied. Additionally, the two calculated dose distributions on the CBCT and planCT were imported to the myQA software and cross‐compared.

### 2.4 Workflow validation

#### 2.4.1 Ethos on‐phantom contouring

As contouring strongly affects the feasibility and reproducibility of the QA workflow, the performance of Ethos auto‐contouring on the pelvis insert inside the SRS phantom was evaluated. To this end, auto‐contouring on the original full pelvis CT, insert‐only CT and insert inside the SRS phantom CT/CBCT was qualitatively compared. To further evaluate the Ethos auto‐contouring performance on the CBCT, the planCT* contours were intentionally modified to anatomically misaligned shapes, such as protrusions and overlap of body, prostate, rectum and bladder contours.

#### 2.4.2 Gamma analysis

To validate the feasibility and practicality of the proposed end‐to‐end QA workflow, the initial setup and steps described in Section 2.3 were repeatedly executed. Initially, six consecutive adaptations of the plan created in step B of the initial setup were performed over three days. Small contour variations were introduced, reflecting the inherent intra‐observer variability between each fraction that naturally occurs when contouring real patient anatomy in clinical practice. Accordingly, the MUs of the adapted plans ranged from 1105 to 1118 MUs, differing only slightly from the 1103 MUs of the original plan. The calculated and measured dose distributions were compared in the myQA software using Gamma Analysis. Global, absolute GPRs were calculated for clinically relevant criteria, such as 3%/2 mm, 2%/2 mm, 3%/1 mm and 2%/1 mm. A low‐dose threshold of 10% was applied and the 100% dose normalization was defined as the maximum dose of the measurement in all cases. Subsequently, the Confidence limit was calculated, following the methodology described in the AAPM Task Group 119 report, using the formula:

(1) $$\mathrm{Confidence}\;\mathrm{Limit}\;\left(\%\right)=100\;\%-\left(100\;\%-\mathrm{mean}\right)+1.96\;\sigma$$

where mean represents the average value and σ the standard deviation (SD) of each criterion's GPR.

Additionally, specific plan parameters of the adapted plan, such as gantry and collimator angle (−1°, −3°, −5°, −7°, −9° relative to the original angles) and MU/plan (+1%, +3%, +5%, +7%, +9% relative to the original MU values) were manually modified, irradiated, and measured. The calculated dose distribution of the original adapted plan and the measured dose distribution of each irradiated altered plan were compared by calculating the GPR for the aforementioned criteria. To simulate a misalignment between kV imaging and MV irradiation, a positioning error of 3 mm caudal, anterior, and lateral right of the detector was intentionally introduced by a couch shift after the initial alignment with the laser. To investigate the effects of erroneously applying an inappropriate preset for the pelvis area, several CBCT presets deviating from the 125 kV pelvis preset were acquired, including pelvis large (140 kV), head (100 kV) and image gently (80 kV). During Ethos HyperSight commissioning for clinical use, the HU‐to‐density calibration curve for 125 kV had been acquired and validated. The use of a different acquisition preset results in variations in reconstructed HU values, leading to corresponding differences in the assigned densities. Each corresponding calculated adapted plan was irradiated and measured. To investigate the potential influence of differing contouring on each CBCT, the Dice Similarity Coefficient (DSC) and Hausdorff Distance (HD) were calculated by comparing each planning target volume (PTV) with the PTV on the 125 kV pelvis CBCT. For each criterion, the GPRs of all flawed plans were compared with the corresponding calculated Confidence limit derived from the six non‐flawed adapted plans. This comparison enabled assessment of whether each flawed plan was detectable.

To assess the magnitude of the resulting dose perturbation and dosimetric impact, dose‐volume histogram (DVH) parameters for the modified plans were calculated as a percentage deviation from the original adapted plan in Eclipse using D98% for the PTV and V95% for organs at risk (OARs—rectum and bladder).

## 3 RESULTS

### 3.1 Ethos on‐phantom contouring

While Ethos successfully auto‐contoured the original full pelvic CT, it failed to generate contours on the planCT when the pelvic insert was within the SRS phantom.

Notably (though outside the scope of the presented workflow), contouring was also successful on a cropped pelvic insert DICOM dataset, which included no phantom structure and undefined surrounding pixel values outside the insert. Auto‐contouring repeatedly failed in the planCT, which included either the phantom or air around the insert. It is plausible that auto‐contouring works on human anatomy including cropped images, which it was developed for, but fails when unexpected pixel values or textures are present in the vicinity.

Figure 5a illustrates the deformed contours (shifted 10 mm cranial) of the planCT in red and the auto‐generated contours produced by Ethos ETP on the non‐deformed pelvis insert anatomy of the CBCT in yellow on both the planCT* (deformed, Figure 5a—left) and the CBCT (non‐deformed, Figure 5a—right). The Ethos auto‐contouring did not accurately delineate the anatomical structures of the anthropomorphic insert visible on the CBCT. Instead, it primarily produced rigidly propagated contours of the planCT*.

**FIGURE 5:**
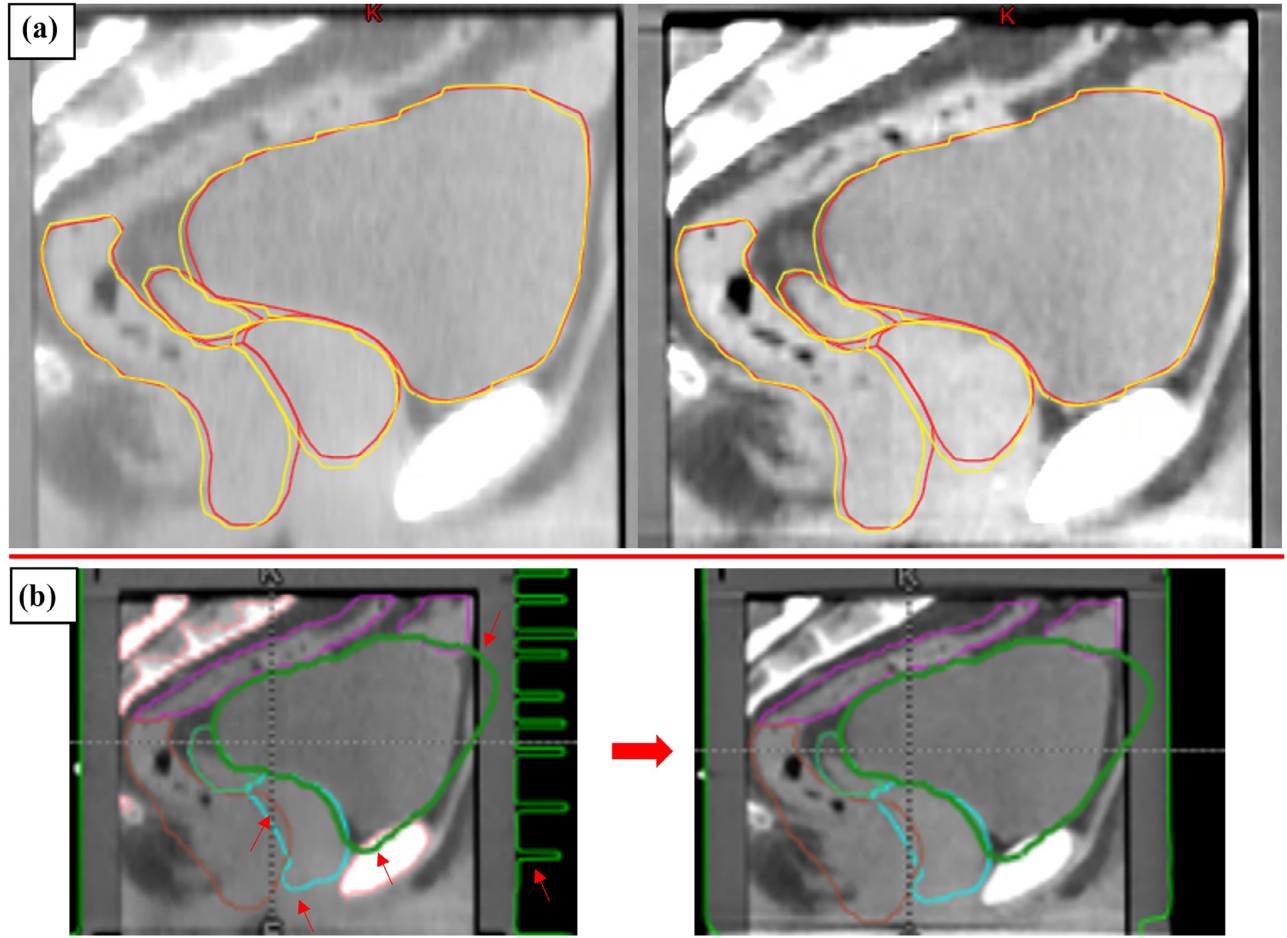
(a) Comparison of deformed contours on planCT* (10 mm cranial shift, red) with contours on the CBCT of the non‐deformed insert (yellow), shown on the planCT* (left) and the CBCT (right). (b) Intentionally modified, misaligned contours on the planCT (left) rigidly propagated to the CBCT (right).

Against the expectation of being displaced 10 mm caudal from the deformed contours, both contour sets coincided for the most part. Figure 5b demonstrates that intentionally modified, anatomically misaligned contours on the planCT* (Figure 5b—left), excluding the body contour, were rigidly propagated to the CBCT (Figure 5b—right).

### 3.2 Gamma analysis

Figure 6 presents a two‐dimensional, pixel‐based representation of global gamma values using the 3%/2 mm criterion and 10% low‐dose threshold for three example adaptive plans from a total of six non‐flawed plans. All gamma grids show high agreement between the calculated and measured values, indicated by predominantly low (blue) gamma pixel values, which is consistent with the GPRs calculated for each criterion.

**FIGURE 6:**
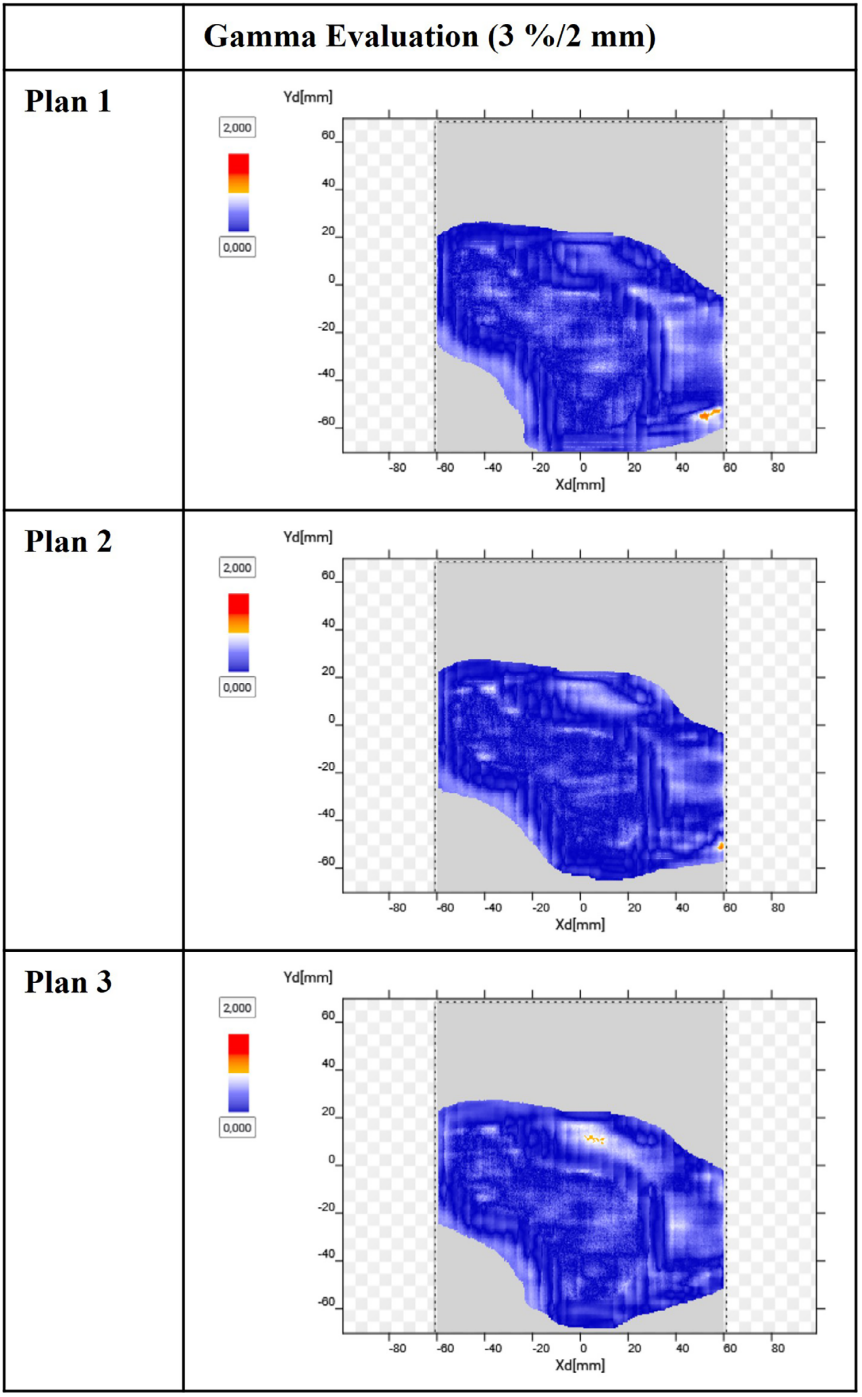
Global gamma maps for three example adaptive plans using the 3%/2 mm criterion and 10% low‐dose threshold. The pixel color indicates the gamma value, with blue representing low values and red representing high values.

A cross‐comparison of both dose distributions calculated on the CBCT and planCT demonstrated high gamma pass rates 3%/2 mm > 99%. Additionally, HU comparison between CBCT and planCT showed good agreement. Consequently, this approach provides a reliable framework for validating dose distributions calculated directly on session CBCT imaging.

The Confidence limit for each criterion was calculated using Equation (1) and is listed in Table 1. These limits were then applied to assess the detectability of flawed plans, as shown in Table 2.

**TABLE 1:** GPRs for six adaptive workflow iterations, including mean value, standard deviation (SD) and Confidence limit.

	3%/2 mm	2%/2 mm	3%/1 mm	2%/1 mm
Plan 1	99.8	99.1	99.1	97.0
Plan 2	99.9	99.5	99.8	97.7
Plan 3	99.9	98.2	99.5	95.6
Plan 4	99.9	99.6	97.2	93.9
Plan 5	100.0	100.0	97.7	95.9
Plan 6	100.0	99.7	99.3	96.5
Mean	99.9	99.4	98.8	96.1
SD	0.1	0.6	1.1	1.3
Confidence limit	99.8	98.1	96.7	93.5

**TABLE 2:** GPRs of flawed adapted plans compared with the calculated Confidence limits, defining threshold‐based detectability.

		GPR (%)				DVH parameter	
Changed parameter	Magnitude of change	3%/2 mm	2%/2 mm	3%/1 mm	2%/1 mm	PTV‐D98% (|Δ%|)	OAR‐V95% (max |Δ%|)
		**99.8**	**98.1**	**96.7**	**93.5**		
Gantry	1°	100.0	99.0	99.2	96.4	0.1	1.4
Gantry	3°	99.8	99.0	98.5	95.1	1.2	5.5
Gantry	5°	97.6	95.7	94.3	89.8	3.3	10.6
Gantry	7°	92.1	88.6	87.1	81.5	7.1	17.0
Gantry	9°	84.3	80.2	78.5	72.5	12.7	24.8
Collimator	1°	99.9	98.4	98.2	94.3	0.5	0.0
Collimator	3°	98.1	95.4	93.6	87.4	2.8	5.6
Collimator	5°	92.8	87.8	83.8	74.4	6.4	16.7
Collimator	7°	84.0	77.0	72.1	61.3	11.8	25.9
Collimator	9°	73.3	63.6	60.8	51.7	18.1	35.2
MU	1%	100.0	98.4	98.7	93.6	1.0	5.6
MU	3%	93.2	74.0	83.9	60.1	3.1	14.8
MU	5%	68.8	60.1	55.9	42.3	5.3	24.1
MU	7%	59.2	52.1	42.6	31.2	7.5	31.5
MU	9%	51.6	47.0	32.7	25.8	9.9	40.7
Cranial	3 mm	81.6	74.7	59.9	51.7	7.1	55.6
Anterior	3 mm	79.1	72.7	66.1	57.3	10.1	38.9
Right	3 mm	91.2	87.5	84.8	78.6	4.9	2.3
CBCT	140 kV (pelvis large)	98.5	96.8	92.3	87.4	2.5	3.8
CBCT	100 kV (head)	93.1	90.9	82.6	76.6	23.1	10.8
CBCT	80 kV (image gently)	96.4	93.5	83.9	77.1	12.7	8.6

*Note*: Highlighted in blue indicates that a flawed plan remained undetectable (above threshold). Corresponding DVH parameters are given as the percentage deviation from original plan. The bold numbers (99.8, 98.1, 96.7, 93.5) are reference GPR numbers (the Confidence limit).

Table 2 summarizes how modifications to plan parameters affect GPRs across different evaluation criteria. The Confidence limits, calculated for each criterion from six non‐flawed adaptive plans, serve as thresholds for determining error detectability. A flawed plan was considered undetectable (highlighted blue in Table 2) if its GPR remained above the Confidence limit and detectable (not highlighted in Table 2) if its GPR fell below the corresponding Confidence limit. Larger deviations, such as 5°–9° gantry angle changes, 3°–9° collimator angle changes, and 3%–9% MU variations, were consistently detectable across all criteria. In contrast, smaller modifications, including 1° and 3° gantry angle shifts, 1° collimator adjustments, and 1% MU variation, remained undetectable regardless of the criterion applied. A 3 mm detector misalignment produced noticeably low GPRs and was clearly detectable by all criteria. Table 2 also presents GPRs obtained by comparing a calculated adapted plan based on the 125 kV pelvis preset with measured adapted plans generated from various CBCT presets using the pelvis insert. Selecting an inappropriate preset produced markedly lower GPRs, leading to clear and consistent detectability across all criteria. Analysis of DSC and HD values indicated only minimal variation in PTV contours among all CBCT acquisitions, confirming that differences in GPRs were not influenced by PTV inconsistencies: Across all CBCT presets, the DSC was consistently 0.99, while the HD ranged from 0.92  to 1.37 mm.

Minor modifications of gantry and collimator angles, as well as MUs, resulted in small dosimetric impact on both the PTV and OARs. For undetected flawed plans, D98 % for the PTV percentage deviations remained at or below 1.2%, while V95% for the OARs were at or below 5.6%. In contrast, larger deviations led to progressively greater percentage differences, greater than or equal to 2.8% for the PTV and 5.6% for OARs, correlating with the magnitude of the modification. Detector misalignments and variations in CBCT presets produced D98% and V95% percentage deviations correlating with the magnitude of each observed GPR values.

## 4 DISCUSSION

The proposed end‐to‐end QA workflow is feasible in routine clinical implementation and can be carried out efficiently by a single user. The selected phantom and measurement equipment demonstrated capability for evaluating the entire adaptive workflow, from image acquisition and contouring to plan adaptation, dose calculation, and delivery. However, the assessment of contouring was limited by the missing capability of the Ethos software to auto‐contour the insert anatomy. High‐resolution measurements offered robust validation of the workflow and ensured reliable error detection. After the initial one‐time creation of the datasets (patients), only the equipment must be set up before each workflow execution. With this preparation, the workflow can be performed reproducibly and within a timeframe comparable to that of a standard patient treatment session. However, the one‐time creation of patients in the Eclipse TPS, along with the necessity to manually transfer the RTPlan to Eclipse and then reimport it into the respective patients for dose calculation, proved cumbersome. No simpler workaround was available to streamline this data handling, largely due to the standardized patient and data management structure within the Ethos system. For regular use of the proposed end‐to‐end‐test, the amount of required data transfer can be reduced by generating a verification phantom in Ethos. Performing the gamma analysis in step 3 within the dedicated myQA software also required additional data transfer.

The pelvis insert provided realistic kV images of the included anthropomorphic structures but was not tissue‐equivalent in terms of MV dosimetry, whereby dose measurements were not performed inside the insert itself. The insert's width was approximately 2 mm smaller than the SRS phantom slot, which resulted in a loose fit, mitigated by adding small spacers. The overall SRS phantom and insert geometry deviated substantially from the overall human anatomy, as the anthropomorphic insert is surrounded by 10 cm of water‐equivalent material. Since the Ethos auto‐contouring is trained on human anatomy, auto‐contouring could not be performed accurately for this artificial phantom geometry. The anatomical structures of the anthropomorphic insert acquired by CBCT were not contoured correctly by the Ethos auto‐contouring but were instead merely rigidly propagated contours of the planCT*. It was further observed that when no auto‐contouring was performed on the planning CT, none occurred on the HyperSight CBCT either. Consequently, manual contouring is required to capture anatomical structures for the adaptive workflow. Manual contouring of the organs, particularly the prostate, proved challenging and time‐consuming due to the limited soft‐tissue contrast and consequently indistinct anatomical boundaries. Since a comparison between “ground‐truth” contours on the CBCT anatomy and manually delineated contours would have primarily reflected human contouring variability rather than the performance of the Ethos auto‐contouring system, the contouring evaluation was limited to a qualitative assessment. As contour propagation is a critical risk in oART, the inability to evaluate the system's auto‐contouring limits the clinical generalizability of this specific workflow component. Another limitation related to the phantom and insert geometry is that dose measurement and calculation are performed in a homogeneous medium. However, this assumption is not entirely unrealistic for the pelvic region. To facilitate reproducible manual contouring, a pelvis insert for the SRS phantom featuring artificially delineated organ boundaries would be beneficial. In general, a phantom with a larger slot providing more space for realistic anatomy might permit usable auto‐contouring, though this is not applicable with the fixed slot dimensions of the SRS phantom and increases costs. Furthermore, while detector array measurements provide a valuable QA baseline, the integration of an ionization chamber or other point detectors for absolute dose measurements into future phantom inserts is highly desirable to enable independent, absolute point‐dose verification while accounting for potential dose‐to‐medium conversion complexities.

While only a single virtual deformation scenario was evaluated in this study for validation purposes, future work should investigate a broader range of deformations to fully explore the tool's capabilities.

As a common metric for assessing QA results, the GPR served as the primary measure of workflow validity in this study. It is utilized strictly for threshold‐based detection and does not imply formal statistical testing. To evaluate efficiency and feasibility, the end‐to‐end workflow was repeatedly executed six times with different adaptations while the Ethos system was in routine clinical operation, without errors or system changes. GPRs for various criteria were calculated, evaluated and compared with literature values, demonstrating both high agreement and consistency. The calculated GPR values either fell within or exceeded the literature GPR ranges:^18^, ^19^, ^20^, ^21^, ^22^ 3%/2 mm: 95%–99%, 2%/2 mm: 85%–95%, 3%/1 mm: 95%–99%, 2%/1 mm: 80%–90%. Deviations from 100%, particularly under stricter criteria, likely reflected several factors: At first, the Ethos Acuros XB model itself shows small inherent variations.^23^ Secondly, minor uncertainties in aligning the phantom with the Ethos room lasers might introduce submillimeter isocenter shifts. In order to preserve workflow integrity, no manual post‐alignment was applied to match calculated and measured dose distribution. Differences in spatial resolution introduced uncertainty because the detector acquires data at 0.4 mm resolution, whereas the finest dose‐grid spacing in Ethos is 1.25 mm.

When evaluating GPRs for intentionally flawed plans created by parameter alterations, applying the calculated Confidence limit demonstrated consistent detectability of errors across all criteria. All modified plans produced GPRs below the Confidence limit, except for small adjustments, specifically 1° and 3° changes in gantry or collimator angle and a 1% MU change. These findings align with the clinically applied Varian Ethos tolerances of ± 1.0° for both gantry and collimator angles. Minor modifications resulted in small changes in relevant DVH parameters, whereas progressively larger parameter alterations led to correspondingly larger dosimetric deviations. The proposed workflow demonstrated the ability to detect the larger perturbations evaluated in this study. Undetected modifications resulted in dose deviations that were sufficiently small to fall within typically accepted clinical tolerances. Conversely, detected flawed plans were generally associated with clinically relevant dose deviations. It should be noted that this validation was performed for a prostate case with a rather symmetrical PTV, where small angular deviations are less likely to cause notable dose degradation within the target volume. Adaptive‐specific failure modes, such as incorrect contour propagation or target and OAR deformation, could not be evaluated in the present study due to limitations of the auto‐contouring and the simplified phantom anatomy. Future studies using a more realistic phantom incorporating complete anatomical structures could enable systematic evaluation of these adaptive‐specific failure modes within the proposed QA workflow.

Despite the overall consistency across criteria, stricter criteria such as 2%/1 mm produced more pronounced drops below the Confidence limit. For the measurement equipment used in this study, the calculated Confidence limit being above 93.5% for all tested criteria proved to be a stricter threshold than the TG‐218 universal action limit of 90% or the universal tolerance limit of 95%.^20^ Under the 3%/2 mm criterion, 8 flawed plans still exceeded both universal limits. For example, plans with a 7° gantry angle error, a 5° collimator angle error, a 3% MU deviation, or an incorrect CBCT preset exceeded these universal thresholds, whereas the Confidence limit correctly identified them as flawed. Our use of plan‐specific thresholds represents a deliberate departure from traditional population‐wide TG‐119 baselines and generalized TG‐218 criteria. By calculating confidence limits from six deliveries, we established a constancy check tailored to this specific workflow geometry. This approach yields action limits that are stricter than standard TG‐218 recommendations, allowing for highly sensitive detection of deviations unique to this adaptive scenario.

An explicit threshold to determine plan acceptance under clinical conditions cannot be defined within the scope of this study, as the proposed QA workflow must first be integrated into routine clinical QA to accumulate longitudinal data. This would then enable the establishment of internal clinical threshold levels and facilitate the identification of the most suitable evaluation criteria. The probability of error occurrence and the extent to which such errors might be detected by other QA methods were not considered in this workflow validation. In the case of this study, the focus was solely on whether and to what extent potential errors could be detected by the calculated GPR combined with the calculated Confidence limit and thus the end‐to‐end workflow.

A limitation of the present study is the reliance on a 2D gamma analysis with global normalization and reduced sensitivity to localized dosimetric discrepancies for validation. While 2D measurement‐based approaches remain practical in clinical routine due to their efficiency and robustness, they inherently offer a simplified representation of complex dose distributions. Errors in 3D anatomy or target coverage, such as variations in PTV or absolute dose deviations to OARs, cannot be fully quantified. A 2D gamma analysis is valuable as an initial screening tool to detect deviations. However, to comprehensively evaluate clinical dose impacts under complex error scenarios, full 3D dose reconstructions or volumetric measurements are required. Given the wide‐spread use and availability of 2D arrays, the implementation of an end‐to‐end test with the available equipment amended by only an anthropomorphic insert provides a feasible solution to most centers. Repeated measurements of a plan while incrementally rotating the detector plane may provide quasi 3D information. However, advanced 3D dose reconstruction methods, particularly those based on Monte Carlo algorithms, enable a more comprehensive volumetric assessment. Therefore, a combination of 2D measurement techniques and 3D computational approaches may represent a balanced strategy.

In summary, the proposed workflow introduces a novel end‐to‐end QA method of the oART process, characterized by high‐resolution 2D dose measurements and the use of interchangeable inserts. Its main limitation lies in the integration with the Ethos system, which operates as a standalone platform and therefore requires several manual data‐handling steps especially during initial setup of the test.

## 5 CONCLUSION

The workflow presented in this study proved to be a reliable and feasible end‐to‐end QA workflow for oART with the Varian Ethos system. The results demonstrated that the planning CT can be replaced by a virtually deformed CT allowing the assessment of inter‐fractional changes with one single static insert. Repeated execution of the workflow yielded consistently high GPR values across various criteria, confirming the robustness and reliability of the setup and the results. Deliberate error introduction demonstrated that flawed plans could be reliably detected through high‐resolution measurement, with the GPR consistently reflecting these deviations across all applied criteria. When compared against the calculated Confidence limit, the GPR values of flawed plans fell below this threshold, thereby confirming the detectability of all but the smallest errors. Comparison with DVH parameters for PTV and OARs showed good agreement between technical detectability and the dosimetric impact of flawed plans, emphasizing the low dosimetric impact of small errors. A limitation of the workflow was the absence of auto‐contouring for the insert anatomy and the numerous manual import steps required during the analysis, which limits a quick implementation.

## AUTHOR CONTRIBUTIONS

Julian Niedling, Robert Schindhelm and Sonja Wegener designed the study. Julian Niedling did most of the data collection and analysis. Luis Cervantes generated data required for the study. Julian Niedling wrote the first draft of the manuscript. All authors discussed the study, reviewed the manuscript and agree with publication.

## CONFLICT OF INTEREST STATEMENT

The authors declare, that a patent application covering the described method has been filed by IBA Dosimetry on November 12, 2025. The authors have no personal financial interests related to this patent.

## ETHICAL APPROVAL

This work involves exclusively phantoms, QA equipment and simulated data and did not involve human participants or animal subjects. Formal ethical approval and informed consent were not required for this study.

## Data Availability

Authors will share data upon request to the corresponding author.
